# Advancing cancer therapeutics: new perspectives on mechanisms, regulatory and policy challenges, and innovative multi-modality strategies for the early detection of cardiotoxicity

**DOI:** 10.1007/s11239-026-03282-8

**Published:** 2026-06-10

**Authors:** Richard C. Becker

**Affiliations:** University of Cincinnati Cancer Center, Cincinnati, OH 45267 USA

**Keywords:** Cancer therapeutics, Cardiotoxicity, Regulatory processes, Public–private partnerships

## Abstract

Recent advances in cancer therapeutics, particularly targeted agents and tissue agnostic immunotherapies, have transformed care while introducing new challenges in cardiovascular safety. Although clinical guidelines have evolved, regulatory processes critical to ensuring safe innovation remain under-leveraged and often disconnected from real-world implementation. This gap is especially concerning amid federal downsizing, which has led to significant staff reductions at the United States Food and Drug Administration (FDA) and other health agencies. To meet growing needs in patient safety and therapeutic oversight, a new paradigm is emerging-one that is collaborative, adaptive, and inclusive of all stakeholders. Drug manufacturers, oncologists, cardiologists, clinical trialists, regulators, and technology developers must integrate around shared goals. Cardiovascular endpoints should be integrated into pre–new drug application (NDA) studies, with trial designs that include at-risk populations and robust cardiac monitoring. Early detection tools and mitigation strategies should be co-developed when feasible alongside therapeutics and considered part of the approval process.

**Graphical abstract:**

**Figure:**
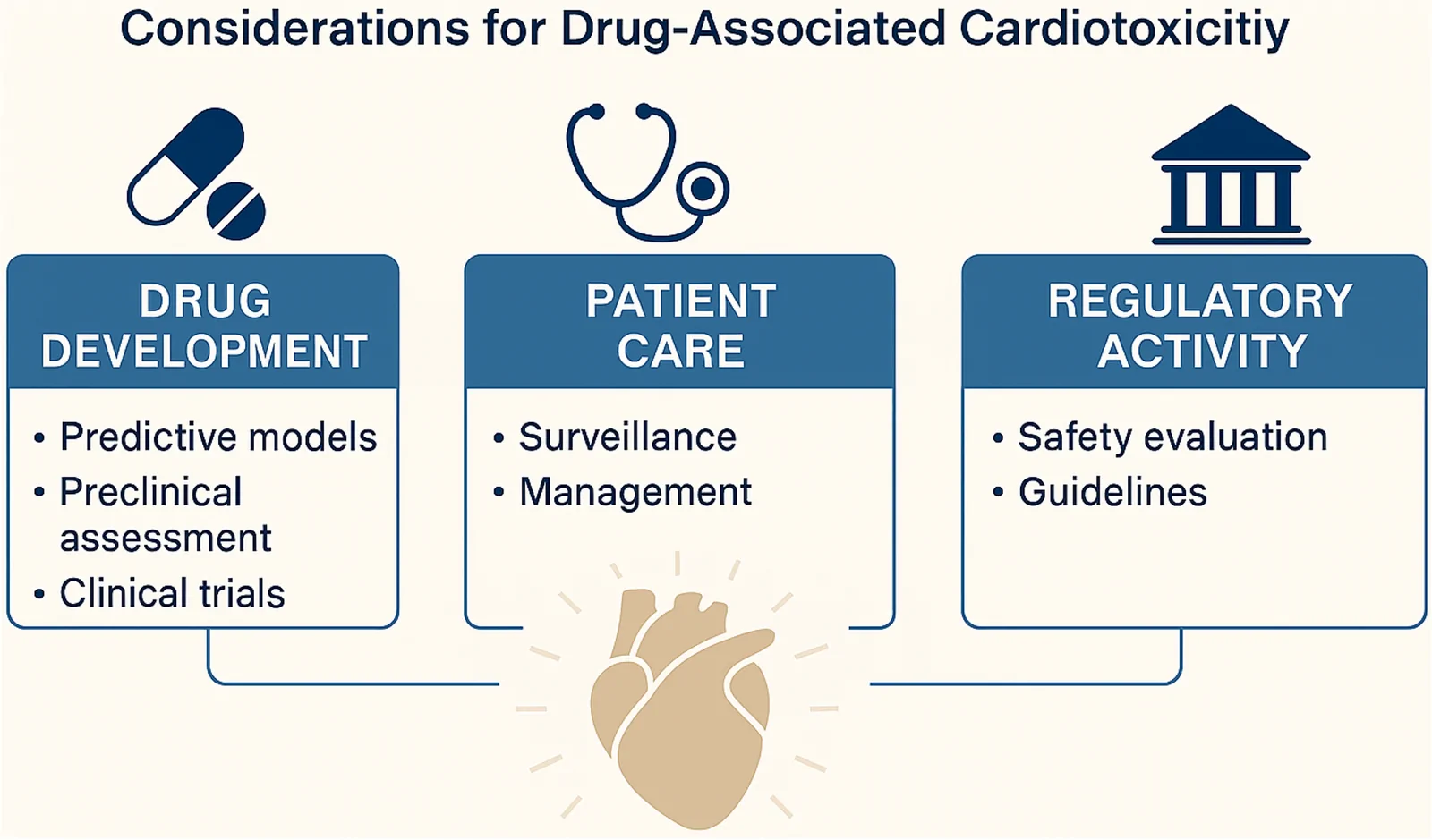

Integrated framework for drug-associated cardiotoxicity across the therapeutic lifecycle. The illustration highlights three essential domains: (1) Drug development, including predictive modeling, preclinical assessment, and structured clinical trials; (2) Patient care, incorporating surveillance and management strategies to mitigate cardiotoxicity risk; and (3) Regulatory activity, emphasizing safety evaluation and guideline development. This framework reflects the multidimensional process required to detect, manage, and prevent cardiotoxicity associated with cancer therapeutics.

## Highlights

- Cardiotoxicity is underrecognized in early-phase cancer drug trials, often emerging 3–5 years post-approval.
- Cardiotoxicity may be seen early in treatment or decades afterward.
- Expedited regulatory pathways often prioritize efficacy over cardiovascular safety, creating gaps in early risk detection.
- Regulatory austerity in the United States Food and Drug Administration may threaten timely and thorough new drug development safety.
- Public–private partnerships are essential to integrate cardiovascular endpoints, biomarkers, and imaging into drug development.
- Artificial intelligence and real-world data offer promising tools for early cardiotoxicity signal detection and risk stratification.
- Policy alignment and multidisciplinary collaboration are critical to ensure cancer therapeutics are effective but do not compromise long-term cardiovascular health.

Cardiotoxicity is an increasing challenge in oncology, influencing both patient outcomes and therapeutic decision-making. The full range of cardiovascular effects from cancer and its treatment may not be fully appreciated by oncologists, cardiologists, public health experts, insurers, and patients. The true impact is summarized below:

Cardiovascular toxicity from cancer treatments presents several unique challenges compared to other organ toxicities:

- **Prevalence:** While many chemotherapeutic agents can harm the liver or kidneys, cardiac toxicity-especially heart failure or cardiomyopathy is also common. Estimates suggest up to 20% of adult survivors who receive cancer therapy develop some form of heart-related dysfunction, with 7–10% experiencing clinically significant cardiomyopathy or heart failure [1].
- **Seriousness & Long-Term Consequences:** Survivors face chronically elevated cardiovascular risk. Rates of heart failure/cardiomyopathy, thrombosis, and myocarditis/pericarditis remain higher with hazard ratios of 1.08, 1.50, and 1.30, respectively compared to the general population, even five years post-treatment. The responsible mechanisms are an area of active investigation. One potential mechanism may be related to clonal hematopoiesis [2].
- **Impact on Treatment:** Cardiotoxicity-especially acute cardiac dysfunction-frequently necessitates modification, interruption, or discontinuation of cancer therapy to prevent irreversible damage. This outcome is more likely than with hepatic or renal toxicities, which are often mitigated with dose adjustments or supportive measures [3].
- **Long-Term Disease Burden:** Beyond immediate treatment effects, survivors have nearly twice the risk of cardiovascular mortality relative to the general population. Conditions like congestive heart failure, ischemia, arrhythmias, and hypertension often manifest years or even decades later [4, 5].

This three-part primer provides a comprehensive exploration of the clinical, regulatory, and technological dimensions of cardiotoxicity associated with cancer therapeutics.

## Part 1 – Clinical spectrum and mechanistic insights into cardiotoxicity from cancer therapies

**Fig. 1:**
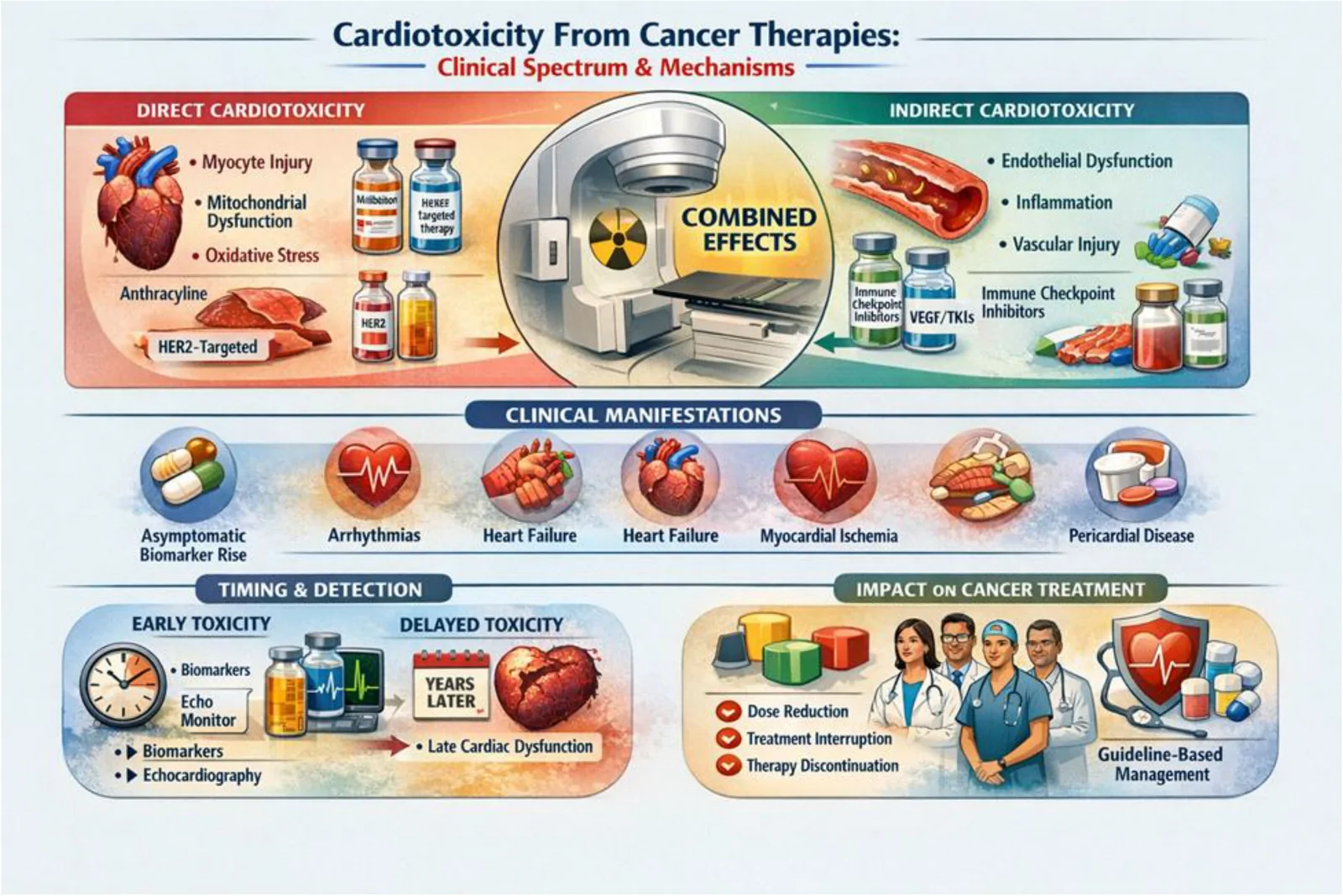
Overview of mechanisms and clinical spectrum of cardiotoxicity. Schematic summary of major biological mechanisms underlying drug‑induced cardiotoxicity and their clinical manifestations, ranging from subclinical myocardial injury to overt heart failure and arrhythmias across the treatment continuum

This article examines the definitions, classifications, and clinical manifestations of cardiotoxicity, highlighting detection methods, timing, incidence, and grading systems. It also addresses real-world data and the impact of cardiotoxicity on cancer treatment decisions (Fig. 1).

## Part 2 – Regulatory and policy challenges for cardiotoxicity detection in oncology drug development

**Fig. 2:**
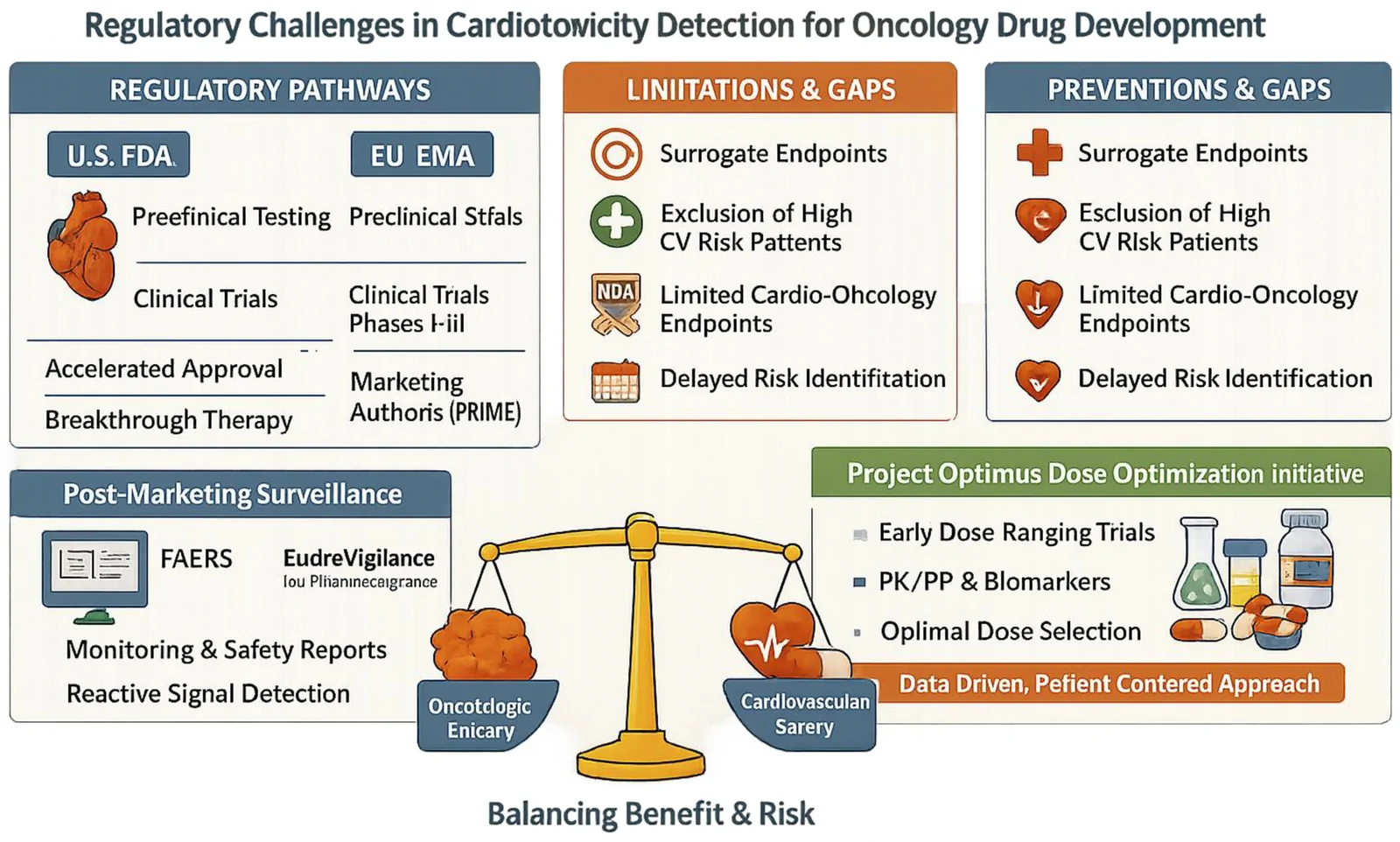
Detection and risk stratification of cardiotoxicity. Conceptual framework illustrates current and emerging approaches for cardiotoxicity detection, including baseline risk assessment, imaging and biomarker surveillance, and integration of temporal changes to improve early identification of cardiac injury

Focusing on regulatory frameworks, this article analyzes current approval processes, pharmacovigilance systems, and the implications of resource constraints within agencies. It explores policy foundations, the role of the FDA Oncology Center of Excellence, and opportunities for public–private partnerships to strengthen oversight and safety (Fig. 2).

## Part 3– Innovative strategies for early detection of cardiotoxicity: artificial intelligence and multi-modality collaborative models

**Fig. 3:**
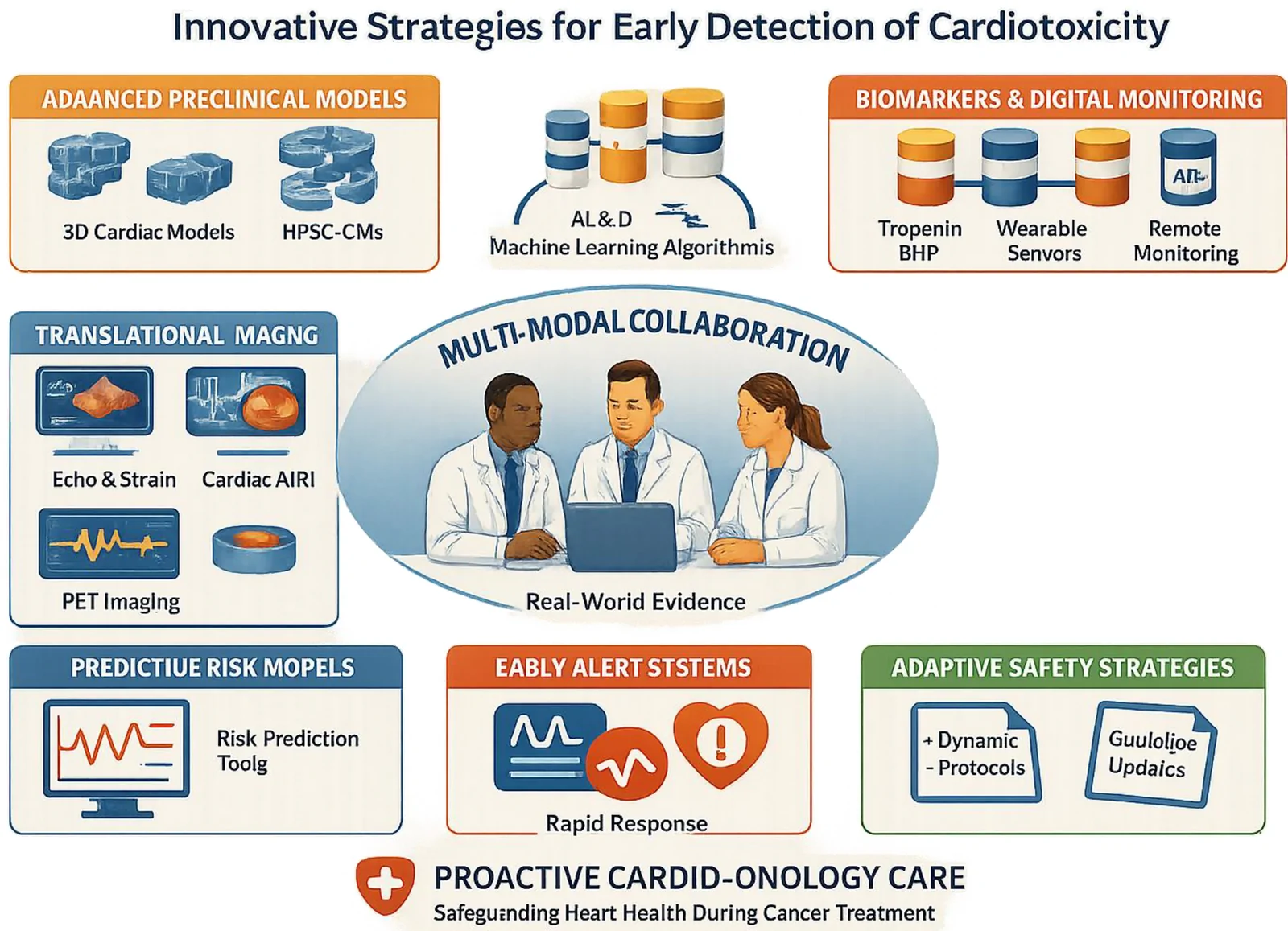
Translational and regulatory pathways for cardiotoxicity mitigation. Integrated model highlighting opportunities to reduce cardiotoxicity through preclinical modeling, clinical monitoring strategies, and regulatory decision‑making, emphasizing feedback between drug development, clinical care, and post‑marketing surveillance

The final article looks ahead to emerging solutions, including advanced preclinical models, imaging, biomarkers, artificial intelligence, and big data capabilities. It emphasizes decentralized surveillance, real-world evidence, integration of regulatory, manufacturer, and health system data, and leveraging global registry information to accelerate detection and response (Fig. 3).

Together, this three-part collection aims to advance understanding of cardiotoxicity and stimulate discussion on optimal strategies and policies for rapid, comprehensive detection during drug development, approval, and post-marketing and clinical use. By aligning drug development with public health priorities and embedding safety intelligence into clinical workflows, this multi-sector approach can reduce the near- and long-term effects of cardiotoxicity in cancer care, ushering in a more responsive and resilient framework for drug safety.

## Introduction

Over the past 2 decades, cancer drug development has undergone a transformative evolution, marked by a surge in approvals of novel therapies, particularly targeted biologics and precision medicines. Lung, breast, and colorectal cancers have been the most common areas for new drug approvals, reflecting both high disease burden and therapeutic innovation. Despite these advances, the rapid pace of development has introduced challenges, especially in cardiovascular safety [6–8].

Cardiotoxicity is a major concern in oncology practice [9]. The data show clearly that cardiotoxicity has increased- possibly with the introduction of new therapeutics, including immunotherapies, however, the basis is multifactorial:

### Rising incidence and detection

-
  - As cancer survival continues to improve, more patients are exposed to cardiotoxic therapies, increasing the cumulative burden of cardiac side effects—particularly among long-term survivors [10].
  - Enhanced surveillance-using advanced imaging (e.g., strain echocardiography, cardiac MRI) and biomarkers (troponin, BNP) identify more subclinical and chronic cardiac events.

### Specific drugs with high cardiovascular-risk

-
  - Anthracyclines (e.g. doxorubicin): Can cause acute irreversible myocardial injury through oxidative stress or a dilated cardiomyopathy decades after treatment.
  - HER2-targeted agents (e.g. trastuzumab): Induce reversible left ventricular dysfunction via disrupted HER2 signaling—especially when combined with anthracyclines.
  - Tyrosine kinase inhibitors, VEGF inhibitors, and antibody–drug conjugates: Linked to hypertension, arrhythmias, and heart failure [11].
  - Immunotherapies (e.g. checkpoint inhibitors) and CAR-T therapies [12]: Associated with myocarditis and inflammatory cardiotoxic effects.

### Effects of aggressive combination therapies

-
  - Combining multiple cardiotoxic agents- such as anthracyclines with HER2-targeted drugs or radiation significantly raises the risk, either through additive or synergistic mechanisms.

### Growing burden of patient risk factors

-
  - Cancer patients, including cancer survivors increasingly have pre-existing cardiovascular risk factors including age, hypertension, diabetes, dyslipidemia, and prior heart disease [13].
  - The intersection of oncologic and cardiovascular risk amplifies susceptibility to cardiotoxic events [14–16].

Pivotal clinical trials often underestimate the incidence of adverse cardiovascular events, while post-approval surveillance reveals significantly higher rates. This discrepancy highlights the limitations of traditional trial designs and underscores the need for more robust safety assessments throughout the drug cycle. The implications are profound, affecting clinical decision-making, patient outcomes, and in some cases survivorship [17].

The escalating cost of cancer drug development, driven by high research expenditures, regulatory complexities, and market dynamics has led to financial strains for patients, reimbursement pressures for payers, and pricing scrutiny for manufacturers. Stakeholders across the spectrum are converging around a shared goal- to deliver effective, safe, and economically viable treatments [18].

Regulatory agencies such as the United States Food and Drug Administration (FDA) and European Medicines Agency (EMA) have introduced expedited pathways (e.g., Fast Track, Breakthrough Therapy, Accelerated Approval, Conditional Marketing Authorization) to speed access to promising therapies. While these mechanisms have improved availability of drugs, they often rely on surrogate endpoints and offer proportionately limited safety data, leaving risks insufficiently characterized [19]. Although frameworks exist to strengthen pre-approval requirements, progress has lagged the pace of drug approvals and the upward trajectory for new cancer therapeutics is higher than ever [20].

Given the rising incidence and clinical detection of cardiotoxicity, impact of near- and long-term outcomes, down-sizing of regulatory agencies, and the inherent limitations of current regulatory and clinical models, there is a pressing need to assess cardiovascular safety earlier, preferably from the outset of drug development [21]. Strategies such as improved preclinical models, incorporation of cardiovascular endpoints in early-phase trials, inclusion of patients with pre-existing cardiovascular disease, predictive modeling based on drug mechanism(s) of action, longitudinal cardiotoxicity registries, and targeted artificial intelligence (AI) applications are essential to ensure that new therapies are both effective and safe [22].

Central to this effort is a collaborative mindset -one that unites public institutions, academic research organizations, national and international cardiology and oncology societies, private industry, including contract research organizations, and regulatory agencies. Such public–private partnerships (PPP) are not only essential for advancing science and innovation but also for safeguarding patient health [23]. Last, there are unmet needs and gaps in the current approach to cardiotoxicity that can be addressed and filled, respectively, complementing substantial efforts from respected societies, organizations, and associations [24–33] that have not sufficiently emphasized drug regulatory processes and the pivotal role of PPP whose roles are expanding exponentially worldwide.

This *cardiotoxicity primer* is designed to provide a structured, three-part exploration of the clinical spectrum, regulatory challenges, and innovative strategies for early detection of cardiovascular toxicity from cancer therapeutics. Its intent is to advance understanding and inform optimal strategies and policies for rapid, comprehensive detection during drug development and clinical use.

**Figure:**
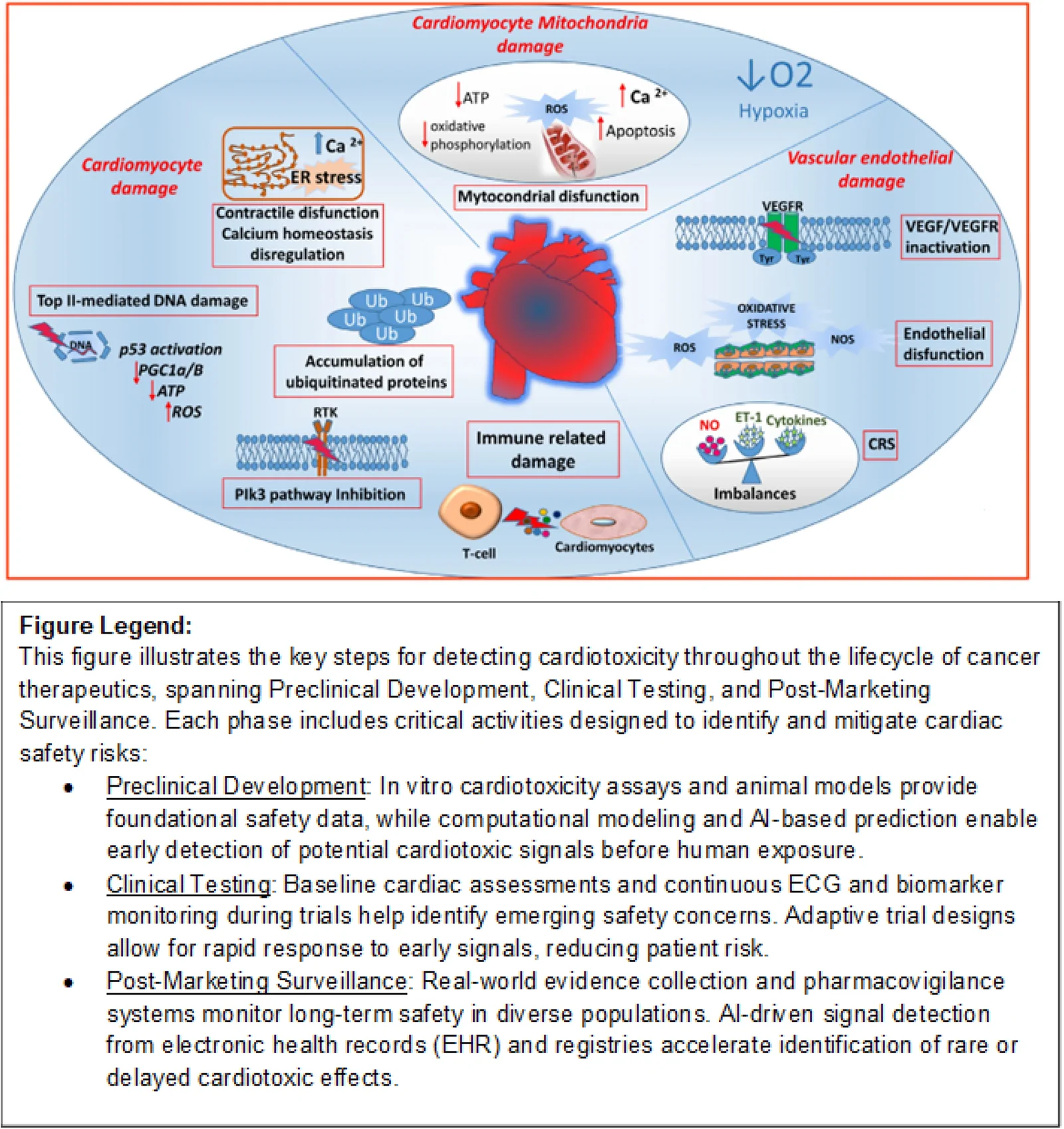

## Data Availability

No datasets were generated or analysed during the current study.
